# The role of acceleration and jerk in perception of above-threshold surge motion

**DOI:** 10.1007/s00221-020-05745-7

**Published:** 2020-02-14

**Authors:** Ksander N. de Winkel, Florian Soyka, Heinrich H. Bülthoff

**Affiliations:** Max Planck Institute for Biological Cybernetics, Max-Planck-Ring 14, 72076 Tübingen, Baden-Württemberg Germany

**Keywords:** Jerk, Acceleration, Perception, Vestibular, Otoliths

## Abstract

Inertial motions may be defined in terms of acceleration and jerk, the time-derivative of acceleration. We investigated the relative contributions of these characteristics to the perceived intensity of motions. Participants were seated on a high-fidelity motion platform, and presented with 25 above-threshold 1 s forward (surge) motions that had acceleration values ranging between 0.5 and 2.5 $\text {m/s}^2$ and jerks between 20 and 60 $\text {m/s}^3$, in five steps each. Participants performed two tasks: a magnitude estimation task, where they provided subjective ratings of motion intensity for each motion, and a two-interval forced choice task, where they provided judgments on which motion of a pair was more intense, for all possible combinations of the above motion profiles. Analysis of the data shows that responses on both tasks may be explained by a single model, and that this model should include acceleration only. The finding that perceived motion intensity depends on acceleration only appears inconsistent with previous findings. We show that this discrepancy can be explained by considering the frequency content of the motions, and demonstrate that a linear time-invariant systems model of the otoliths and subsequent processing can account for the present data as well as for previous findings.

**Electronic supplementary material:**

The online version of this article (10.1007/s00221-020-05745-7) contains supplementary material, which is available to authorized users.

## Introduction

Perception of self-motion results from interactions between different sensory modalities (Howard 1982): the visual system registers optic flow and uses this information to estimate velocity and motion direction (de Winkel et al. 2018), and the vestibular system and a variety of somatosensory (proprioceptive/kinesthetic and tactile) sensors throughout the body transduce the magnitude and direction of inertial stimulation directly (Zaichik et al. 1999).

For translational motion, which is the focus of the present study, the otolith organs of the vestibular system are generally regarded as the primary contributor (Walsh 1961; Valko et al. 2012). Physiological studies performed in monkeys (Fernandez and Goldberg 1976; Massot et al. 2011; Yu et al. 2012; Jamali et al. 2013; Laurens et al. 2017) have shown that the output of the otolith organs, the afferent neural firing rate, and subsequent processing of these signals depends on acceleration and jerk (rate of change of acceleration), and changes with frequency content. Similarly, psychophysical studies performed in humans show that motion direction detection thresholds (Benson et al. 1986; Soyka et al. 2009, 2011), absolute detection thresholds (Heerspink et al. 2005) and differential thresholds (Grant and Haycock 2008) depend on acceleration and jerk. In the majority of these psychophysical studies, motion perception is described as a linear time-invariant (LTI) system, which characterizes the system’s output in response to sinusoidal acceleration inputs using transfer functions. Grant and Haycock (2008) took a somewhat different approach: they presented subjects with trapezoidal motion profiles, and characterized these motions in terms of their peak acceleration and jerk values. They then quantified the relative contributions of these properties to perceived motion intensity. Consistent with the literature, the results indicated that perceived motion intensity depended both on acceleration and jerk. However, the range of acceleration and jerk values used was small, and the experimental paradigm consisted of a relatively small number of pairwise comparisons. Consequently, it is not known to what extent these findings can be generalized.

The present study was designed to expand upon these results: participants were presented with a larger range of motion stimuli, with acceleration and jerk values approximately an order of magnitude larger than in the Grant and Haycock study (Grant and Haycock 2008), using a high-fidelity motion simulator. In addition to a pairwise comparison (2-interval forced choice, 2IFC) task, participants also performed a magnitude estimation (ME) task. The relative contributions of acceleration and jerk were determined by using a statistical model of perception that simultaneously accounts for the data in the 2IFC and ME tasks. Previous work considering LTI models focused on perception around the absolute detection threshold. Here, we also assessed whether an LTI model can predict perception for supra-threshold motion. By combining the data of these different tasks and analyses, we aimed to contribute to a better understanding of perceived motion intensity.

## Methods

### Ethics statement

The experiment was performed in accordance with the Declaration of Helsinki. Participants provided written informed consent before participation in the study. The experiment protocol was approved by the ethical committee of the Eberhard Karls University in Tübingen, Germany (reference: 355/2019BO1).

### Participants

Seven participants (mean age 33.3, SD 14.0, 4 females) were recruited for the study. Three of them were employees of the Max Planck Institute for Biological Cybernetics; the remaining four were recruited from the institute participant pool. Fitness to participate in a simulator study was assessed by questionnaire. Participants were informed of the experimental goals and procedures in compliance with the notion of informed consent. External participants were compensated for their time at a rate of $\EUR {8}$/h.

### Setup

Stimuli were presented using an eMotion 1500 hexapod motion system (Bosch Rexroth AG, Lohr am Main, Germany) available in our laboratory (Nesti et al. 2017; de Winkel et al. 2017, 2018). The platform was controlled using Simulink software (The MathWorks, Inc., Natick, MA, USA). Participants were seated in an automotive style bucket seat (RECARO GmbH, Stuttgart, Germany) that was mounted on top of the platform. Participants were secured in the seat with a five-point safety harness (SCHROTH Safety Products GmbH, Arnsberg, Germany). To minimize head movements, participants wore a Philadelphia-type cervical collar. Actuator noise was masked by having participants wear earplugs with a 37 dB signal-to-noise ratio (UVEX Arbeitsschutz GmbH, Fürth, Germany) as well as a wireless headset (Plantronics, Santa Cruz, California, United States) that provided active outside noise cancellation and played white noise during stimulus presentation.

### Stimuli and tasks

**Fig. 1:**
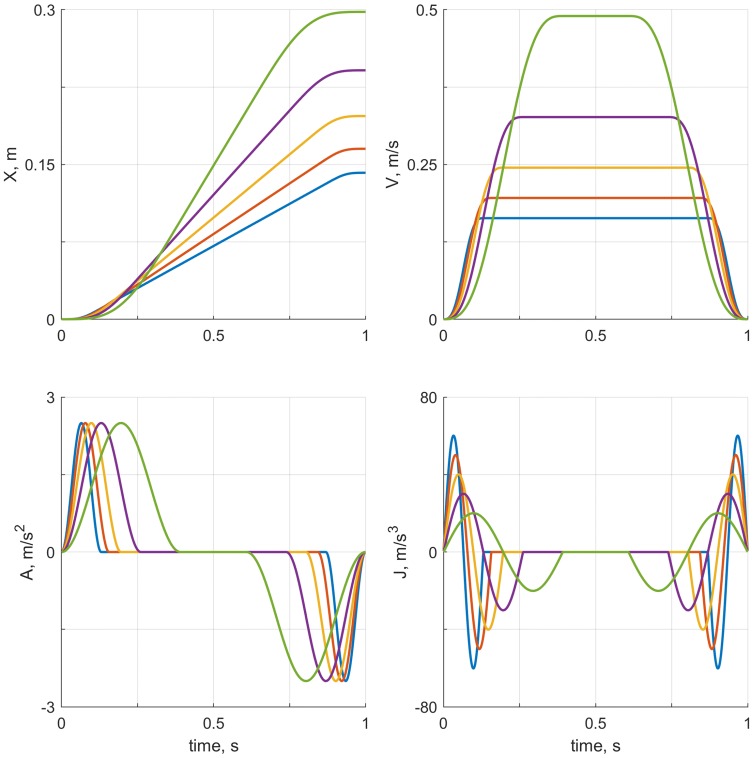
Visualizations of the motion profile in terms of position *X* (upper left), velocity V (upper right), acceleration A (lower left), and jerk J (lower right), for motions with a maximum acceleration $A_\text {max}$ of $2.5\text {m/s}^2$, and maximum jerks $J_\text {max}$ of $[20, 30, 40, 50, 60]\ \text {m/s}^3$. The properties of different motions are matched between panels on the basis of their color

To determine the relative contribution of acceleration and jerk to perceived motion intensity, we created 25 motions. These motions were 1 s forward translations (surge motions), consisting of an acceleration phase, a constant velocity phase, and a deceleration phase. The acceleration phase was defined as $A_t = A_{\text {max}}\sin ^2(\pi t /t_1)$. $t_1$ was varied to achieve different combinations of acceleration and jerk within individual motions: there were five levels of maximum acceleration ($A_\text {max}=[0.5, 1.0, 1.5, 2.0, 2.5]\text {m/s}^2$) and five levels of jerk ($J_\text {max}=[20, 30, 40, 50, 60]\text {m/s}^3$), resulting in 25 different motion profiles. As an illustration, the five motion profiles for the highest acceleration level ($2.5\;\text {m/s}^2$) and each jerk level are shown in Fig. 1.

**Fig. 2:**
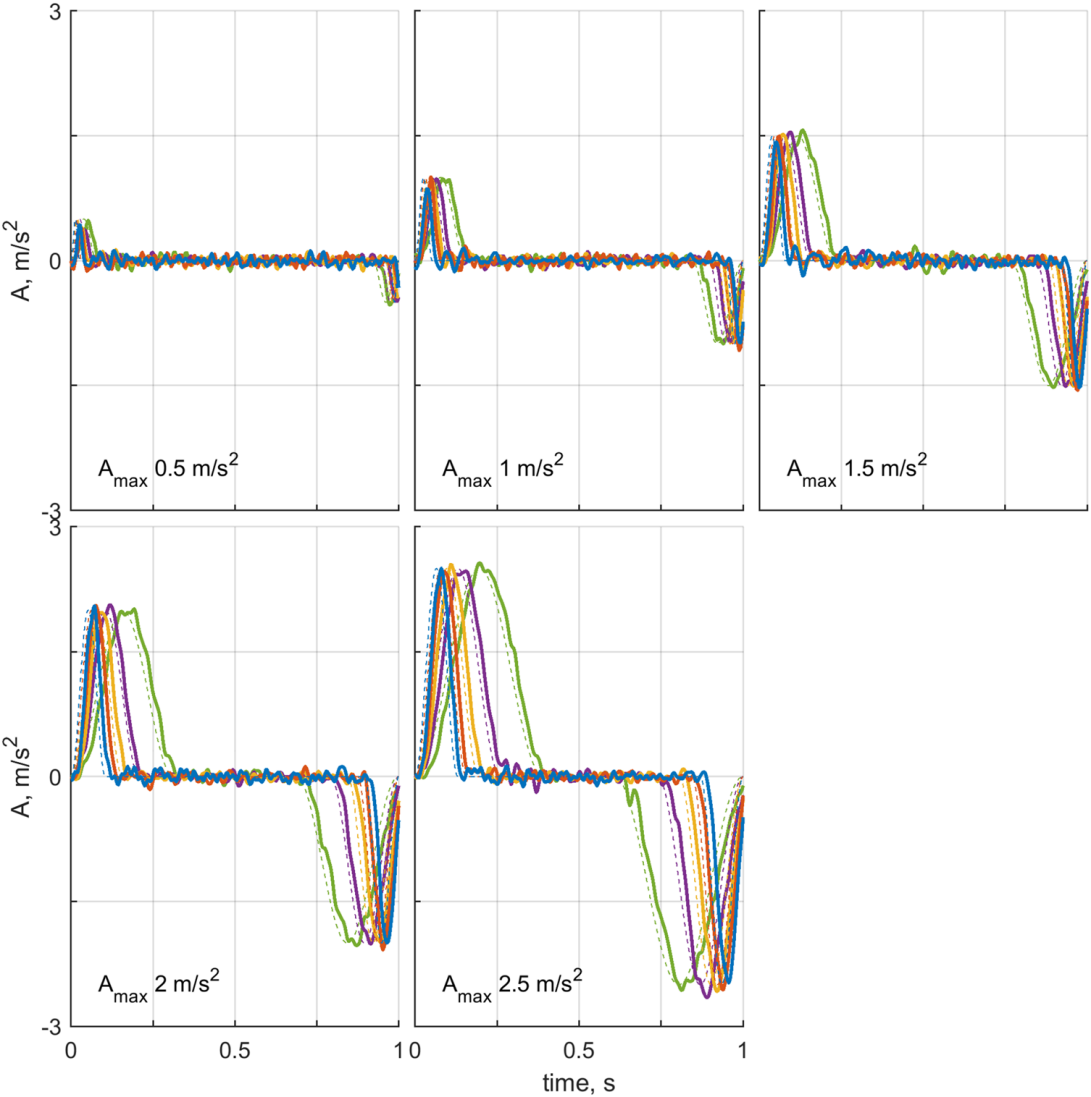
Accelerometer recordings for all motion profiles. Each panel shows the recordings for a single acceleration level (as noted); the five maximum jerks $J_\text {max}$ of $[20, 30, 40, 50, 60]\ \text {m/s}^3$ are represented by the colors green, purple, yellow, orange, and blue, respectively. The corresponding measured mean (standard deviation) peak acceleration values are: 0.46(0.06), 1.01(0.04), 1.54(0.02), 2.04(0.03), and 2.57(0.06) $\text {m/s}^2$. Thick lines show the recordings, and thin dashed lines the commanded motions. The signals are low-pass filtered using a third-order Butterworth filter with a cutoff frequency of 40 Hz to filter out electrical interference

The ability of the platform to accurately reproduce the motion profiles was tested by comparing the commanded motion to actual accelerations, recorded using an accelerometer. The recordings indicated that the platform reproduced the motion profiles accurately (see Fig. 2).

The motions were used in two tasks: a magnitude estimation (ME) task and a two-interval forced choice task (2IFC). To block visual cues, which would provide additional information on velocity (Howard 1982; Pretto et al. 2009), participants performed both tasks with their eyes closed.

*ME task* In the ME task, participants were asked to attribute intensity ratings to the motions, using an interval measurement scale (Stevens et al. 1946). Participants were presented with a motion after which they provided a response, and then were moved back to the initial position. Participants first completed three practice trials. On these trials, the motion with the middle acceleration and jerk levels (i.e., $1.5\;\text {m/s}^2, 40\;\text {m/s}^3$) was presented, which they were told to attribute the value ‘100’. This value served as a reference for subsequent motions: for instance, a motion feeling twice as strong should be attributed twice the reference value. After the practice trials were completed, participants were presented with each of the 25 motions in a random order. The ME task took about 5 min to complete.

The ME task was always performed after the 2IFC task. This was done to be sure participants were familiar with the range of motions, without explicitly informing them of the range of motions and thereby potentially truncating their responses.

*2IFC task* In the 2IFC task, participants performed pairwise comparisons on 300 experimental trials. To generate the trials, we first formed pairs of the 25 different motion stimuli that were defined. These pairs of motions were generated using the MATLABnchoosek function, which gives all the possible combinations of drawing 2 items out of 25 items $(25!/2!(25-2)!=300)$.

Second, we randomized the order of the motions within the pairs. This was necessary because the MATLAB function returned the pairs as an ordered list in which the first motion tended to have larger acceleration/jerk values than the second. This would be problematic because predominantly presenting motions with larger peak accelerations and/or jerks first would bias the responses toward stating that the first motion of a pair was more intense.

**Fig. 3:**
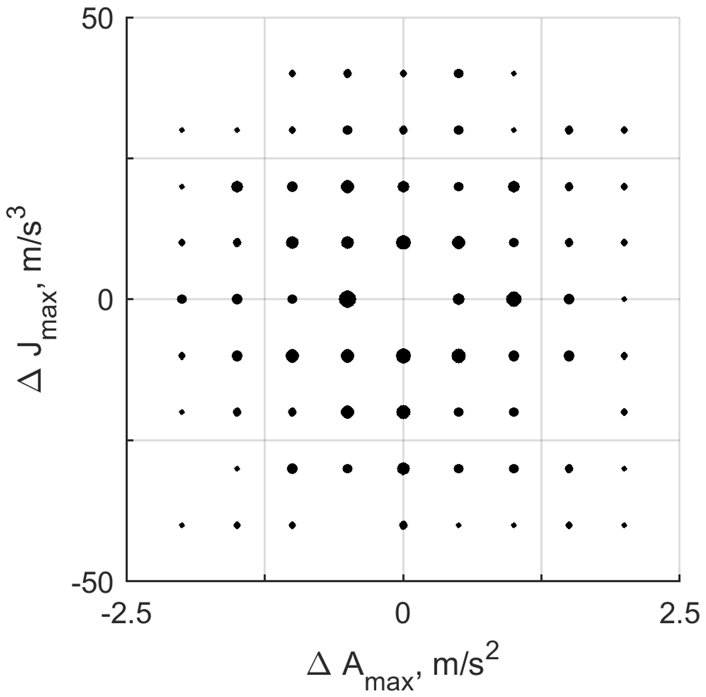
Overview of the conditions presented to an example participant (id 1) in the 2IFC task, and the number of repetitions per condition. The size of the dots corresponds to the number of repetitions. The smallest dots represent 1 repetition; the largest dot represents 14 repetitions

From the five values that peak acceleration could take on and the five for jerk, theoretically nine difference values $\Delta A_\text {max} = A_\text {max}(\text {motion} 2) - A_\text {max}(\text {motion} 1)$, and nine $\Delta J_\text {max} = J_\text {max}(\text {motion} 2) - J_\text {max}(\text {motion} 1)$ can be obtained, ranging from the smallest minus the largest to the largest minus the smallest (e.g., 0.5–2.5 = $-2;$ 2.5–0.5 = 2). Consequently, the $\Delta$ values for the 300 trials are distributed over a $9\times 9$ grid. This distribution is not uniform but peaks around the smaller $\Delta$, because there are relatively more combinations that lead to smaller $\Delta$ values (e.g., 1–0.5 = 0.5; 1.5–1 = 0.5, 2–1.5 = 0.5, 2.5–2  = 0.5, but only 2.5–0.5 = 2). Because of the randomization of the order of motions within the trials, the distribution of $\Delta A_\text {max}$ and $\Delta J_\text {max}$ also differed slightly between participants, and not all points on the grid were presented. This is illustrated in Fig. 3. Finally, we randomized the order of the trials.

Participants initiated each trial themselves by means of a button press. The first motion of a pair was presented 1 s after the trial was initiated; the second motion was presented 2 s after completion of the first. At the end of the trial, participants indicated which motion of the pair was more intense by means of a button press (i.e., ‘first’, or ‘second’). After the response was received, the simulator was moved back to its initial position over 3 s. Including breaks, the 2IFC task took approximately 1.5 h to complete.

*Instructions* The instructions given to participants were formulated to reflect the idea that responses on the tasks are based on intensity percepts, which result from a combination of information on acceleration and jerk. For the ME task, the (written) instructions were: “your task is to provide subjective ratings on the perceived intensity of motion by attributing numbers to stimuli verbally”. For the 2IFC task, the instructions were: “you will be presented with sequences of two motions, and asked to rate which of the two is more intense”. In addition, we provided the following instruction: “try to perform the tasks intuitively: when the motions have stopped, attribute a number or make a judgment on which motion was more intense based on your first impression”. These instructions were deemed sufficient, as the participants did not ask for additional explanations.

### Perception model

We hypothesized that a percept of motion intensity $\psi$ is constructed from observations $A_{\text {max}}^*, J_{\text {max}}^*$ (i.e., internal representations) of the maximum acceleration $A_{\text {max}}$ and jerk $J_{\text {max}}$ for a given motion, and that the contribution of each variable may depend on the value of the other. The latter means that, for instance, the effect of acceleration may be larger for small jerks than it is for large jerks.

We model the percept as a combination of $A_{\text {max}}^*, J_{\text {max}}^*$, and their interaction $A_{\text {max}}^* \times J_{\text {max}}^*$. We also assume that the observations are unbiased and have normally distributed errors. Consequently, the percept is modeled as a normally distributed random variable, with mean $\mu _\psi$ as

1 $$\begin{aligned} \mu _\psi = \omega _{A} A_{\text {max}} + \omega _{J} J_{\text {max}} + \omega _{AJ} (A_{\text {max}}\times J_{\text {max}}) \end{aligned}$$

and variance $\sigma _\psi ^2$. In Eq. (1) above, $\omega _{A}, \omega _{J}, \omega _{AJ}$ are the weights for the observations on acceleration, jerk and their interaction, respectively. In the remainder of the text, we will use the same symbols to refer to coefficients for these effects. It should be noted that stimulus-dependent noise (i.e., Weber’s law) is not included in this model. We tried to fit a version of the model that also included stimulus-dependent noise, but in this case the model fitting routine could not find a unique solution. In the following two sections, we describe how the percept may be transformed into responses on the different tasks.

*ME task* In the ME task, participants have to express their percept verbally, as a number. We assume that this process involves a linear transformation from the perceptual domain to a numerical domain. Using the expressions for the mean and variance of the percept, the probability of a response on the ME task $r_\text {ME}$ is given by

2 $$\begin{aligned} \Pr (r_\text {ME}) = \Phi (r_\text {ME},\mu _\text {ME}, \sigma _\text {ME}) \ , \end{aligned}$$

where $\Phi (\cdot )$ is the normal distribution function, and

3 $$\begin{aligned} \mu _\text {ME}&= K \mu _\psi + r_0 \end{aligned}$$

4 $$\begin{aligned} \sigma _\text {ME}&= \sqrt{K^2 \sigma _\psi ^2} \ . \end{aligned}$$

*K* is the scaling factor from the perceptual domain to the numerical domain; $r_0$ is an intercept. The factor $K^2$ is included in the equation for the standard deviation because when a variable ($\psi$) is scaled by a factor *K*, its variance increases by the square of that factor (Freund 1962).

*2IFC task* In the 2IFC task, the response is the binary outcome of a comparison between the magnitude of two intensity percepts $\psi _a, \psi _b$. We assume that these percepts are independent, and that participants respond positively, namely that the second motion *b* of a pair was more intense than the first *a*, if $\psi _b > \psi _a$. This particular response is coded as $r_\text {2IFC} = 1$; the opposite response is coded $r_\text {2IFC} = 0$.

Consequently, responses reflect the difference between $\psi _b-\psi _a$. When the difference is positive, this means $b>a$; when the difference is negative, $a>b$. Using that the percepts are normal distributed random variables with mean as in Eq. (1) and variance $\sigma _\psi ^2$, their difference is also a normal distributed random variable with mean

5 $$\begin{aligned} \mu _{\text {2IFC}} = \mu _{\psi _b}-\mu _{\psi _a} \end{aligned}$$

and variance and standard deviation as

6 $$\begin{aligned} \sigma _{\text {2IFC}}^2&= \sigma _{\psi _a}^2 + \sigma _{\psi _b}^2 \end{aligned}$$

7 $$\begin{aligned} \sigma _{\text {2IFC}}&= \sqrt{2}\sigma _\psi . \end{aligned}$$

For a given pair of stimuli *a*, *b*, the probability of a positive response is 1 minus the integral over this distribution from $(-\infty ,0]$. This is equivalent to

8 $$\begin{aligned} \Pr (r_{\text {2IFC}} = 1) = \Phi ^{-1}\left( \frac{\mu _{\psi _b}-\mu _{\psi _a}}{\sqrt{2}\sigma _\psi } \right) , \end{aligned}$$

where $\Phi ^{-1}$ is the normal cumulative distribution function, and $\sigma _\psi$ the common noise parameter. Note that this is effectively a probit model (Bliss 1934), which will be used in separate analyses of the data collected in the 2IFC task.

*Model comparisons* To evaluate the performance of the model, we compared its overall fit (referred to as ‘full’) to a number of partial models. These partial models either account for subsets of the data, or include a subset of the effects. Comparing the fit of the partial models to the full model allows us to assess whether it is indeed likely that responses on both tasks result from the same perceptual process, and what this process is. Three comparisons were made: as a first alternative model, we combined individual model fits for the two tasks (referred to as ‘add’). This comparison allows us to evaluate whether participants used the same information/strategy in both tasks. We also compared the fit of the full model to different versions of the perception model: one omitting the interaction term, where perceived intensity is a linear combination of acceleration and jerk, named ‘main’; and one that additionally omits the term for jerk, named ‘acc’, where perception depends on acceleration only. Based on these comparisons, we choose the model that provides the most parsimonious description of participants’ behavior. We used the Bayesian information criterion (BIC) score as the basis for these comparisons (Schwarz 1978).

### Linear time-invariant systems model

As noted in the introduction, much research on the vestibular system has been performed from an (aerospace) engineering perspective; modeling motion perception based on otolith stimulation as a linear time-invariant (LTI) system (Walsh 1961; Fernandez and Goldberg 1976; Benson et al. 1986; Soyka et al. 2009, 2011; Heerspink et al. 2005; Grant and Haycock 2008; Mayne 1974; Hosman and Van der Vaart 1978; Hosman and Stassen 1999). Whereas an in-depth treatment of LTI systems is beyond the scope of the present paper (for an introduction, see for instance: Soyka et al. 2011), we do include an analysis using this method. The purpose of this analysis is to provide a benchmark for comparison between methodologies typical for psychology and engineering. Moreover, the parameters of available LTI models have all been determined from absolute (direction) detection thresholds. Inclusion of this analysis for above-threshold motion thus also serves to validate these models for a novel range of motions.

**Fig. 4:**
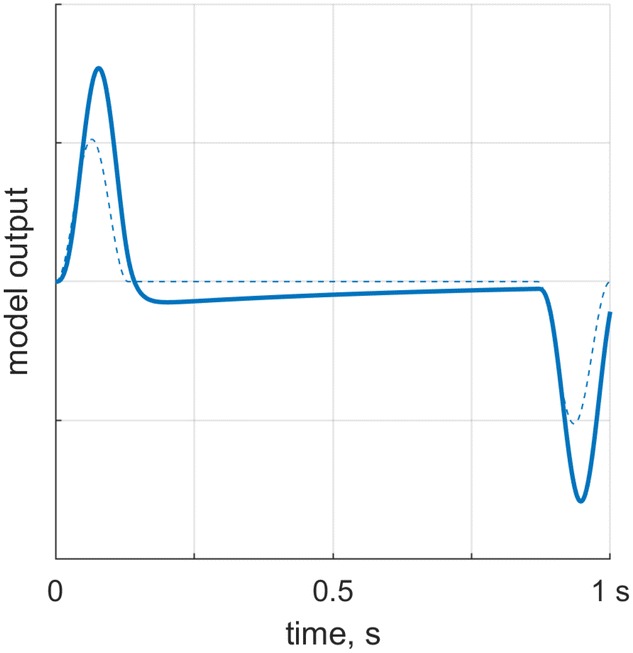
Behavior of the LTI model with parameters found by Soyka et al. (2011). The figure shows the system output (thick line) for one of the motion profiles (thin dashed line). The motion corresponds to the identically colored motion profile in Fig. 1 ($A_{\text {max}}=2.5~\text {m/s}^2$ and $J_{\text {max}}=60~\text {m/s}^3$). Note that the scaling is arbitrary

The LTI model is a transfer function (Eq. 9), which is based on a simplified model of how acceleration inputs bend the sensory hair cells (cilia) of the otoliths, leading to an output that can be interpreted as proportional to a neural firing rate:

9 $$\begin{aligned} H(s) = K \times \frac{(1+\tau _N s)}{(1+\tau _1 s)(1+\tau _2 s)}, \end{aligned}$$

here *s* is the complex number frequency parameter, *K* is a gain, which scales the output, and $\tau _N, \tau _1$ and $\tau _2$ determine how the output signal changes relative to the input signal in terms of frequency content. The behavior of this transfer function using parameters found by Soyka et al. (2011)^1^ is illustrated in Fig. 4. The figure shows the model output for one of the presently used motion profiles according to this transfer function.

## Results

**Fig. 5:**
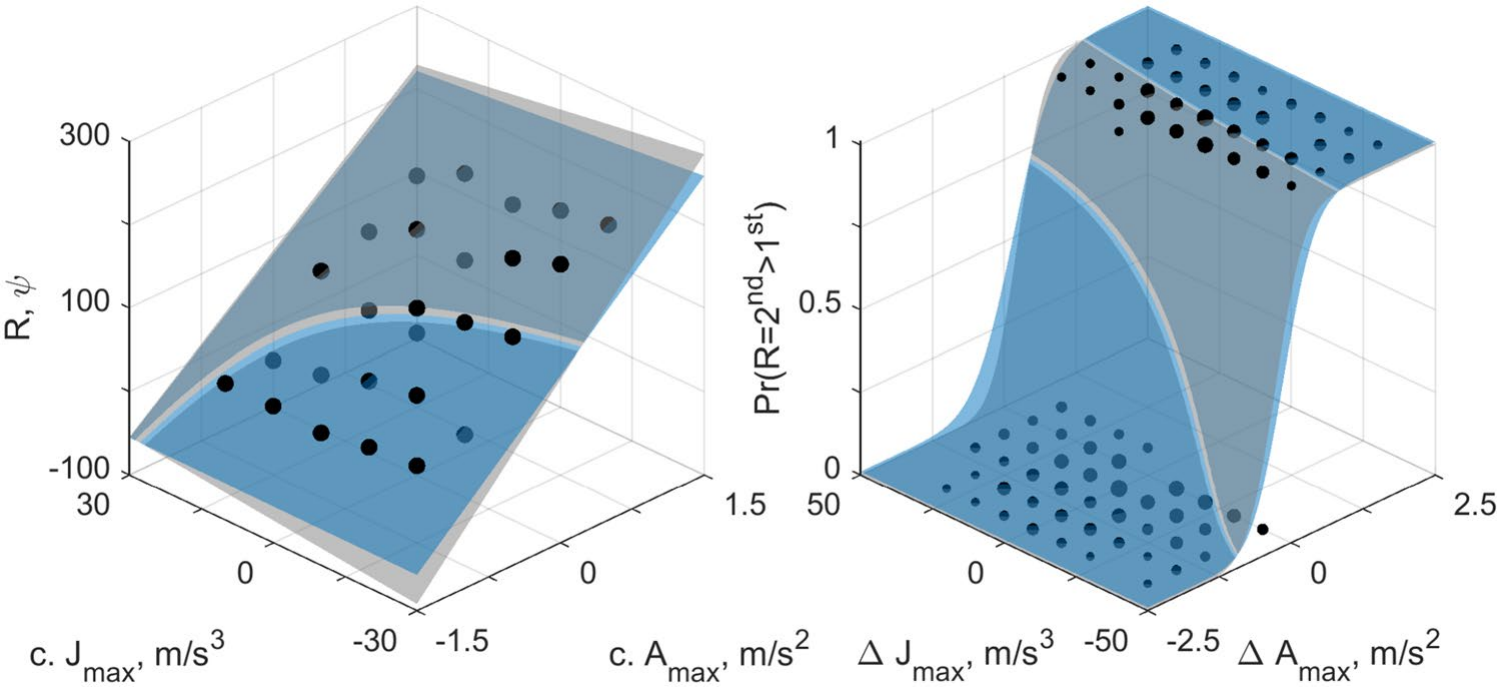
Data and model fits for an example participant (id 1). The left panel shows the findings for the ME-task, and the right panel the findings for the 2IFC-task. Dots represent individual responses. The gray surfaces show the separate model fits (i.e., for the task represented by the panel); blue surfaces show the fit of the joint perception model. For the left panel, the axes $c.A_{\text {max}}, c.J_{\text {max}}$, represent the (centered) peak acceleration and jerk values, respectively. The vertical axis represents responses R in arbitrary units $\psi$. For the right panel, the axes labeled $\Delta A_\text {max}$ and $\Delta J_\text {max}$ show the difference in peak acceleration and jerk values between the second and first motion of a pair. In this panel, the size of the dots is proportional to the corresponding number of observations. The vertical axis represents the probability of responses that the second motion was perceived as more intense than the first

The data from the ME and 2IFC tasks were analyzed separately, using linear models (ME task) and generalized linear models (2IFC task), as well as simultaneously, using the perception model described in the “Perception model” section. As an illustration, the data and model fits are shown for an example participant in Fig. 5. In addition, we compare the present findings to predictions made using an LTI systems model based on the functioning of the otoliths (Soyka et al. 2011). The separate analyses were performed first to evaluate the main and interaction effects of acceleration and jerk in the two tasks independently. Fitting these models is equivalent to separate fits of the perception model to data of the two tasks. By combining the results of the individual fits, an evaluation of the evidence for the combined perception model can be made using a statistical criterion. To account for individual differences, the data from each participant were analyzed individually. Overall conclusions were drawn by combining individual results.

### ME task

Data obtained in the ME task were analyzed using a linear model, which had the following form in Wilkinson notation: (Wilkinson and Rogers 1973)

10 $$\begin{aligned} r_\text {ME} \sim 1 + A_\text {max} + J_\text {max} + A_\text {max} \times J_\text {max}\ . \end{aligned}$$

The ‘1’ term indicates the intercept. All terms on the right hand side of Eq. 10 were centered (i.e., the medians were subtracted from every value) (Hox et al. 2017). The model was fitted to the data using the fitlm function of the MATLAB Statistics and Machine Learning toolbox (MATLAB 2017). Estimated coefficients are presented in Table 1 below. We also present standardized coefficients for acceleration, jerk and their interaction. Note that these can be interpreted as the relative contributions to ratings of perceived motion intensity. Standardized coefficients were calculated as $\omega ^*_i = {\sigma _i\omega _i}/{\sigma _{r_\text {ME}}}$ (see e.g., Long 1997; Hox et al. 2017), where *i* represents acceleration, jerk, or the interaction.

**Table 1:** Unstandardized and standardized parameter estimates for the ME model

id	Unstandardized				Standardized		
	$r_0$	$\omega _{A}$	$\omega _{J}$	$\omega _{AJ}$	$\omega ^*_{A}$	$\omega ^*_{J}$	$\omega ^*_{AJ}$
1	**92.14**	**110.00**	− 0.15	− 0.52	**1.17**	− 0.03	− 0.29
2	**73.71**	**63.72**	− 1.03	0.08	**0.86**	− 0.28	0.05
3	**82.07**	**108.80**	0.41	− **1.06**	**1.37**	0.10	− **0.69**
4	**65.86**	**100.76**	0.89	− **1.18**	**1.54**	0.27	− **0.93**
5	**144.82**	**182.00**	− 0.38	− 1.06	**1.18**	− 0.05	− 0.36
6	**70.93**	**131.72**	0.93	− **1.38**	**1.46**	0.21	− **0.80**
7	**86.07**	**74.00**	− 0.40	− 0.00	**0.95**	− 0.10	0.00
${\bar{x}}$	87.94	110.14	0.04	− 0.73	1.22	0.02	− 0.43

Boldfaced parameters are different from 0 at the $\alpha =0.05$ significance level (assessed by *t* tests). The row labeled ${\bar{x}}$ shows the average value for each parameter

For comparison between the tasks and modeling approaches, we divide the average standardized coefficients by the sum of their absolute values. This yields relative weights ${\tilde{\omega }}_{A}=0.730, {\tilde{\omega }}_{J}=0.012$ and ${\tilde{\omega }}_{AJ}=-0.258$.

The findings suggest that the subjective intensity of motion increases with the size of the acceleration, and that this effect may be moderated by the magnitude of the jerk. This moderation effect means that the strength of the effect of acceleration decreases for larger jerk values.

### 2IFC task

Data obtained in the 2IFC task were analyzed using a generalized linear model, which had the following form in Wilkinson notation (Wilkinson and Rogers 1973)

11 $$\begin{aligned} \Phi ^{-1}(\text {r}_\text {2IFC}) \sim -1 + \Delta A_\text {max} + \Delta J_\text {max} + \Delta ( A_\text {max} \times J_\text {max}) \ , \end{aligned}$$

where $\Phi ^{-1}$ represents the probit link function, ‘$-1$’ indicates the omission of an intercept, and $\Delta A_\text {max}, \Delta J_\text {max}$ represent the difference between the peak acceleration and jerk values of the second and first motion, respectively. All terms on the right hand side of Eq. 11 were centered using the same method as for the ME-task. An intercept was omitted because theoretically, the probability of a positive response should be 0.5 when $\Delta A_\text {max}, \Delta J_\text {max}$ (and their interaction) are 0. The model was fitted to the data using the fitglm function of the MATLAB Statistics and Machine Learning toolbox (MATLAB 2017). Estimated and standardized coefficients are presented in Table 2. Standardized coefficients were calculated as $\omega ^*_i = {\sigma _i\omega _i}/{\sigma _{r_\text {2IFC}}}$ (see e.g., Long 1997; Hox et al. 2017), where *i* represents acceleration, jerk, or the interaction. ${\sigma _{r_\text {2IFC}}}$ had a fixed value of 1.

**Table 2:** Unstandardized and standardized parameter estimates for the 2IFC model

id	Unstandardized			Standardized		
	$\omega _{A}$	$\omega _{J}$	$\omega _{AJ}$	$\omega ^*_{A}$	$\omega ^*_{J}$	$\omega ^*_{AJ}$
1	**2.71**	− 0.01	0.00	**2.77**	− 0.29	0.11
2	**1.80**	− 0.00	0.01	**1.83**	− 0.04	0.32
3	**2.54**	− **0.04**	**0.03**	**2.59**	− **0.88**	**1.35**
4	**3.00**	0.00	− 0.00	**3.07**	0.08	− 0.18
5	**3.64**	0.01	− 0.01	**3.72**	0.13	− 0.53
6	**4.70**	0.01	0.00	**4.80**	0.21	0.08
7	**2.90**	− 0.03	**0.05**	**2.95**	− 0.59	**2.87**
${\bar{x}}$	3.04	− 0.01	0.01	3.11	− 0.20	0.57

Boldfaced parameters are different from 0 at the $\alpha =0.05$ significance level (assessed by *t* tests). The row labeled ${\bar{x}}$ shows the average value for each parameter

For comparison between the tasks and modeling approaches, we divide the average standardized coefficients by the sum of their absolute values. This yields relative weights ${\tilde{\omega }}_{\text {A}}=0.801, {\tilde{\omega }}_{\text {J}}=-0.051$ and ${\tilde{\omega }}_{\text {AJ}}=0.148$.

The findings indicate that the discrimination task was performed predominantly on the basis of the difference in acceleration between motions.

### Perception model

The perception model (“Perception model”) was fitted to the data by minimizing the negative log-likelihood, using the MATLABfmincon function of the Optimization toolbox (MATLAB 2017). Parameters $\omega _A, \omega _J, \omega _{AJ}$ are the weights for the observations on acceleration, jerk, and their interaction; *K* and $r_0$ are the response gain and intercept in the ME task, respectively. The standard deviation for the ME data was treated as a free parameter, and the standard deviation for the 2IFC data was fixed at 1. Consequently, the model can be seen as a combination of the separate analyses with the constraint that the weights for acceleration, jerk and their interaction are the same for the two tasks. Model coefficients (and standardized coefficients for acceleration, jerk, and their interaction) are presented in Table 3.

**Table 3:** Parameter estimates for the perception model

id	Unstandardized					Standardized		
	$\omega _A$	$\omega _J$	$\omega _{AJ}$	*K*	$r_0$	$\omega ^*_A$	$\omega ^*_J$	$\omega ^*_{AJ}$
1	**2.98**	− 0.01	− 0.01	**33.19**	**92.14**	**2.03**	− 0.12	− 0.23
2	**1.83**	− 0.01	0.00	**33.40**	**73.71**	**1.24**	− 0.12	0.16
3	**2.76**	− **0.04**	**0.02**	**18.49**	**82.07**	**1.85**	− **0.53**	**0.69**
4	**3.27**	0.01	− 0.01	**19.31**	**65.86**	**2.23**	0.14	− 0.37
5	**3.70**	0.00	− 0.01	**44.37**	**144.82**	**2.52**	0.04	− 0.43
6	**4.91**	0.01	0.00	**16.01**	**70.93**	**3.34**	0.18	− 0.14
7	**3.07**	− 0.03	**0.05**	**14.22**	**86.07**	**2.09**	− 0.40	**1.62**
${\bar{x}}$	3.22	− 0.01	0.01	25.57	87.94	2.18	− 0.12	0.18

Boldfaced parameters are different from 0 at the $\alpha =0.05$ significance level (assessed by *t* tests). The row labeled ${\bar{x}}$ shows the average value for each parameter

The standardized coefficients were ‘*x*-standardized’, meaning they were calculated as $\omega ^*_i=\omega _i\sigma _{i}$, where the subscript *i* refers to a particular term (Long 1997). The values reported in the table were calculated using the $\sigma$ parameters from the ME task.

For comparison between the tasks and modeling approaches, we divide the average standardized coefficients by the sum of their absolute values. This yields relative weights ${\tilde{\omega }}_{A}=0.879, {\tilde{\omega }}_{J}=-0.047$ and ${\tilde{\omega }}_{AJ}=0.074$. The findings are consistent with the separate analyses, showing that responses are primarily driven by acceleration.

### Model comparisons

We assessed whether the perception model (‘full’) provides a fair account of the data by comparing its fit to the combined fit of the separate models (‘add’). In addition, we compared the fit to a model omitting the interaction effect (‘main’), and to a model additionally omitting the term for jerk (‘acc’). These comparisons were made on the basis of the Bayesian information criterion (BIC). This is a measure of relative model quality based on the model likelihood. The score includes a penalty for the number of parameters (e.g., Hox et al. 2017). Models with a lower score are preferred. The calculated BIC scores are presented in Table 4.

**Table 4:** Model BIC scores

id	BIC			
	add	full	main	acc
1	402.4	397.7	**392.9**	402.4
2	446.2	446.9	441.6	**436.0**
3	**408.0**	412.8	412.9	409.6
4	413.6	415.1	410.7	**405.8**
5	431.0	428.1	**424.6**	426.2
6	383.1	387.4	381.7	**376.9**
7	**340.1**	348.8	362.8	370.3
Overall	2933.3	2918.3	2895.3	**2881.6**

‘add’ represents the combined score of models fit to the data of the two tasks separately, ‘full’ represents the perception model; ‘main’ the model excluding the interaction term and ‘acc’ the model including only a term for acceleration. The ‘overall’ scores were calculated on the basis of the sum of the likelihoods, number of parameters and number of observations. Boldfaced values indicate the best (i.e., lowest) BIC value for each row

For 3/7 participants, we found positive evidence (id 2,4,6; $\Delta \text {BIC}=2-6$) for the acceleration-only model; for another, the evidence for this model and the main-effects model was about equally strong (id 5; $\Delta \text {BIC}=1.6$); and for another participant the evidence for this model was about equally strong as the evidence for the separate fits (i.e., ‘add’: id 3; $\Delta \text {BIC}=1.6$). For the participant with id 1, the best fitting model is the one with main effects, indicating a negative additive effect of jerk; and for the participant with id 7 the best fitting model is the additive model, suggesting this participant may have applied different strategies in the two tasks.

Overall, the model comparisons favor the ‘acc’ model, with $\Delta \text {BIC}=13.7$. A $\Delta \text {BIC}>10$ is considered decisive evidence (Kass and Raftery 1995). This indicates that data of both tasks is best described with a model that only includes acceleration as a predictor.

### Linear time-invariant systems model

To assess whether the LTI model can account for responses on the ME and 2IFC tasks, we processed each motion profile using the transfer function (as shown in Fig. 4). The transfer function was defined using the MATLABtf function, and the motion profiles were passed through the transfer function using the lsim function. These functions are part of the Control System toolbox (MATLAB 2017).

**Fig. 6:**
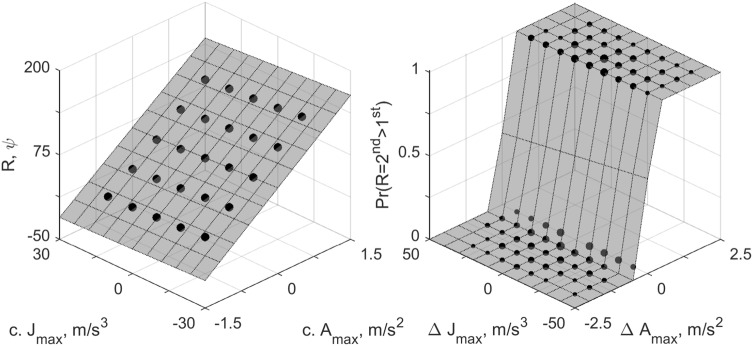
Simulated responses to the ME (left panel) and 2IFC (right panel) tasks generated on the basis of outputs from the LTI model given in Soyka et al. (2011). For comparison with Fig. 5, the same sets of stimuli were used as those presented to participant ‘id 1’. For the right panel, the size of the dots is proportional to the number of simulated responses, which was kept equal to the number of actual observations for the participant

Responses on the ME-task can be generated using the LTI model by taking the peak of the absolute value of the model output for each motion. We can then fit the same (statistical) linear model to these data as was done for the human participants. Similarly, we can generate binary ‘responses’ by comparing the peak values of the LTI model outputs for all different motions, and then fit the probit model to this data. The results of this approach are shown in Fig. 6. Note that this approach does not consider the perceptual noise that is present in actual human data, such that 1. ‘responses’ for the ME task lie on the plane exactly, and 2. there are no mistakes in the 2IFC task, causing the slope of the psychometric function to be steeper than it is for actual participant data.

By comparing Fig. 6 with Fig. 5, it can be seen that the LTI model reproduces the human data quite accurately. Fitting the linear model that was used for the ME task to the simulated data yielded standardized coefficients $\omega ^*_{A}=1.029, \omega ^*_{J}=-0.078, \omega ^*_{AJ}=-0.046$, corresponding to relative weights ${\tilde{\omega }}_{A}=0.893, {\tilde{\omega }}_{J}=-0.068$ and ${\tilde{\omega }}_{AJ}=-0.040$. Fitting the probit model that was used for the 2IFC task yielded standardized coefficients $\omega ^*_{A}=22.477, \omega ^*_{J}=-0.004, \omega ^*_{AJ}=0.010$, corresponding to relative weights ${\tilde{\omega }}_{A}=0.974, {\tilde{\omega }}_{J}=-0.003$ and ${\tilde{\omega }}_{AJ}=0.023$. For this latter procedure it should be noted that because there is no noise in the responses, there is a perfect separation between acceleration/jerk values for which the model predicts zeros and ones. In other words, the fit does not have a sloping area where the probability of a positive response gradually increases; instead, there is a hard cutoff. Nevertheless, the fit shows that where the cutoff lies depends on acceleration only, which is consistent with the results obtained for human participants.

## Discussion

The goal of the present study was to determine the relative contributions of acceleration and jerk to perceived motion intensity. In a separate analysis of the ME task, a positive effect of acceleration was found for all participants, and a negative interaction effect between jerk and acceleration was found for three participants. This latter finding implies that the strength of the effect of acceleration decreases for higher levels of jerk. In terms of relative contributions, perceived intensity was primarily driven by acceleration, with a weight approximately three times larger than the interaction effect. In a separate analysis of the 2IFC task, positive effects of acceleration were again found for all participants, and an opposite, positive, interaction effect was observed for two participants. In terms of the relative contributions, responses were again driven by acceleration, with a weight about five times larger than that of the interaction effect. A joint analysis of the data obtained in both tasks indicated that percepts of motion intensity could in fact be explained with a single model, and that the most parsimonious description is obtained when this model includes acceleration only. During debriefing, some participants indicated that they dissociated an ‘initial kick’ from a subsequent ‘push’, and that their responses were driven by the latter. These introspective accounts of how participants performed the task are consistent with the experimental data and suggest that those observations where the effect of acceleration appeared to be moderated by jerk could have cognitive causes.

**Fig. 7:**
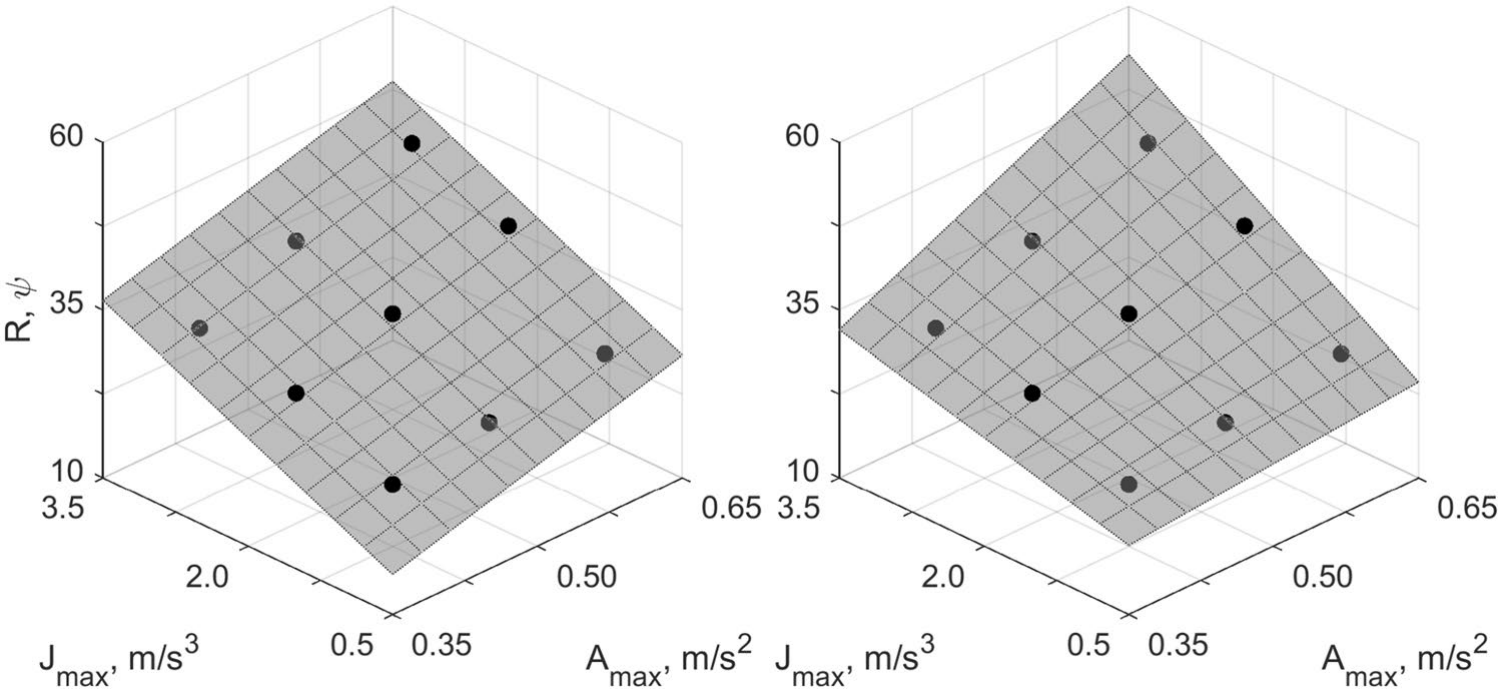
Peak LTI model Soyka et al. (2011) output for motion profiles used in Grant and Haycock (2008) (dots) and fits of statistical models (surfaces). The left panel shows the fit of a model with linear terms for acceleration and jerk only; the right panel shows a model that additionally includes an interaction term. Note how the model in the left panel is a tilted plane, whereas the model on the right shows a distinct curvature, indicative of the interaction effect

To the best of our knowledge, the present research question has only been addressed in a small number of previous studies, using acceleration and jerk levels at the absolute detection threshold. Most of these studies were performed with the aim of modeling perception based on the otoliths as an LTI system. These models provide a simplified account of how the hair cells of the otoliths respond to inertial stimulation. Typically, the parameters of these models are determined from absolute detection thresholds (Mah et al. 1989; Zaichik et al. 1999; Heerspink et al. 2005), or direction detection thresholds (Benson et al. 1986; Soyka et al. 2009, 2011, 2013), and therefore the models indirectly also account for cognitive processes that ultimately result in the responses on the detection tasks. Because we obtained subjective ratings of motion intensity and differential thresholds rather than detection thresholds, and because the present acceleration and jerk levels far exceed the threshold values, the present results cannot be compared with these studies directly. However, by processing the presently used motion stimuli using the LTI model described in Soyka et al. (2011), we were able to simulate responses for both experimental tasks. By subsequently comparing the statistical model for simulated data to the model for actual human data, we can compare the two approaches. Doing so showed that also according to the LTI model, responses should be driven by acceleration. This result appears inconsistent with the results of the only other study where the relative contributions of acceleration and jerk were determined explicitly (Grant and Haycock 2008). The authors of this study designed trapezoidal motion stimuli with peak acceleration and jerk values distributed over a three-by-three rectangular grid, and had participants perform a series of pairwise comparisons for this grid. From the obtained responses, they determined whether and how the probability that participants judged one motion to be more intense than another depended on acceleration and/or jerk, similar to the present 2IFC task. It was reported that there were positive effects of both acceleration and jerk, whereas in the present study responses depended on acceleration only. This apparent inconsistency can be explained by considering the LTI model, as well as the choice of predictors included in the statistical model; whereas the present motions lasted 1 s, and thus had frequency content of 1 Hz and above, their motions lasted approximately 3 s, and thus contained frequencies of $\frac{1}{3}$ Hz and above. According to the LTI model, it is in this range of frequencies that the output changes from being proportional to both acceleration and jerk (0.01–1 Hz) to just acceleration ($>1$ Hz). When we simulate responses for the trapezoidal motion profiles specified in Grant and Haycock (2008) using the LTI model (Soyka et al. 2011), and then fit a regression model with only main effects for acceleration and jerk to this data (Fig. 7, left panel), we find positive coefficients for both ($\omega _{A}=40.92, \omega _{J}=6.80$). These coefficients are very similar to the reported values ($\omega _{A}=6.15, \omega _{J}=0.76$), when expressed in relative terms (simulated: ${\tilde{\omega }}_{A}=0.86, {\tilde{\omega }}_{J}=0.14$; reported ${\tilde{\omega }}_{A}=0.89, {\tilde{\omega }}_{J}=0.11$). However, when we also consider interaction effects (Fig. 7, right panel), the results change considerably, showing a large positive interaction effect, a positive effect for acceleration, and a negative effect for jerk ($\omega ^*_{A}=3.84, \omega ^*_{J}=-2.59, \omega ^*_{AJ}=18.54$; relative weights: ${\tilde{\omega }}_{A}=0.15, {\tilde{\omega }}_{J}=-0.10, {\tilde{\omega }}_{AJ}=0.74$. The absolute relative values do not sum to 1 exactly due to rounding error).

It is interesting to note that the outcome of the simulations did not change for variations of the LTI model with other sets of parameters, namely those found by Soyka et al. (2011) based on the data from Zaichik et al. (1999), Heerspink et al. (2005) and Hosman and Stassen (1999). Variation of the parameters resulted in differences in scaling and slight differences in the shape of the outputs, but the conclusions with respect to the present tasks were identical. The reason for this is that differences between sets of parameters mostly affect low frequencies, whereas the motion profiles used in the present study contained mainly high frequencies.

These findings illustrate that a time-domain perspective on motion perception (i.e., characterizing motions in terms of acceleration or jerk) has limited generalizability. More specifically, to use regression models that take into account peak acceleration and/or jerk to model perception requires the specification of individual models for every possible motion over time, which is clearly not feasible. Adopting a frequency domain perspective offers a more versatile alternative: through the use of the LTI model, perception for a much larger range of motions can be captured with a single model. This argument is supported by the fact that the presently used LTI model can explain our results as well as those of Grant and Haycock (2008), whereas the regression models obtained in these studies are very different.

### Limitations and future work

A common finding in psychophysical research is that perceptual noise increases with stimulus magnitude (i.e., Weber’s Law). In this study, the statistical modeling of the responses in the 2IFC task was equivalent to a probit regression. The regression coefficients together specify how well the alternative decision outcomes can be discriminated; the noise coefficient determines the scaling of the regression coefficients. In other words, the same discrimination performance can be achieved with a set of large regression coefficients with a large noise coefficient, as with a set of small regression coefficients with a small noise coefficient—provided that the relative values are equal. For this reason, it is generally necessary to fix the noise coefficient in probit regressions. We initially intended to determine whether the noise increases with stimulus magnitude by simultaneously fitting the statistical perception model to the data of the 2IFC and the ME task. However, because all participants used different subjective scales, this did not yield consistent results. Consequently, it was not possible to include the effects of stimulus-dependent noise in the modeling. Nevertheless, given that the only variability in responses was along the acceleration dimension (Fig. 5), we believe such effects would not affect the conclusion that, for the present motion stimuli, perception was driven by acceleration.

A possible limitation of the present study is that jerk was manipulated by varying the duration of the acceleration/deceleration peaks. By doing so, velocity (and distance, for that matter) was varied as well (Fig. 1). Theoretically, velocity is not the effective stimulus for the body’s inertial sensors; this motion property can only be estimated more or less directly by the visual system, from optic flow (Howard 1982; Pretto et al. 2009). Because participants were blindfolded, estimates of velocity would necessarily result from integration of the acceleration or jerk signals over time, which implies that these higher derivatives drive perception. Moreover, the overall results show that jerk did not actually affect the responses. Nevertheless, it may be argued that because of their covariation, one cannot disentangle the effects of jerk and velocity using these profiles in cases where effects of jerk could occur, such as for motions with lower-frequency content than in the present study. In future work, it would be possible to avoid this confound by defining motion profiles where the acceleration/deceleration pulses are triangular (Siegmund et al. 2005; Siegmund and Blouin 2009). To test whether doing so would affect the outcome of the present study, we defined a set of 1 s motion profiles (hence with similar frequency content as in the present study) with triangular acceleration/deceleration pulses of 250 ms. We then varied the amplitude and timing of the peak to achieve the same maximum acceleration and jerk values as in the present study, and we processed these motions in the same way as we did for the profiles used by Grant and Haycock (2008). The results of these simulations also showed that perception would depend on acceleration only, with a relative weight for acceleration ${\tilde{\omega }}_{A}=0.999$. These additional simulations are described in detail in the appendix, available as supplementary material.

Another limitation is that it was assumed that the motion commands sent to the platform were identical to the motion of the head. We ensured that the platform accurately reproduced the commanded motion using an accelerometer mounted at the platform itself, and minimized the discrepancy between the motion of the head and the platform by using a cervical collar. Because such collars are not infinitely stiff, it is likely that the motion of the head was not identical to the motion of the platform. However, our characterization of the motion in terms of commanded acceleration/jerk proved sufficiently precise to yield accurate predictions, as was the case for a previous study with a similar paradigm (Grant and Haycock 2008). In future work, an accelerometer could be placed at the head to further improve precision.

Finally, despite the absence of an effect of jerk in the experimental data, introspective reports obtained during debriefing indicated that some participants had noted a manipulation of jerk, but did not use this information to perform the experimental tasks. Perception of jerk is not consistent with the predictions of the LTI model. It could be hypothesized that there are distinct sensations of acceleration and jerk. Such a segregation could for instance serve purposes related to postural control, where sudden disturbances with large jerks require a different response than sustained accelerations. This could be tested in future work by specifically instructing participants to focus on a particular motion characteristic.

### Conclusions

We investigated the perception of above-threshold motions with frequency content above 1 Hz, using two different experimental tasks. Our findings indicate that both tasks were performed on the basis of acceleration and did not depend on jerk. We show that the data collected in the present experiment as well as previous findings for motions with lower frequencies, where jerk was found to play a role, can be described by an LTI systems model that captures otolith output and its subsequent processing.

## Electronic supplementary material

Below is the link to the electronic supplementary material.

Supplementary material 1 (pdf 333 KB)

## Footnotes

1 Apart from the parameters found by Soyka et al. (2011), we also evaluated other sets of parameters, namely those found by Soyka et al. (2011) based on the data from Zaichik et al. (1999), Heerspink et al. (2005) and Hosman and Stassen (1999). This did not qualitatively affect the results (also see “Discussion”).
