# Supplementary material for: The role of acceleration and jerk in perception of above-threshold surge motion
Source: Exp Brain Res. 2020 Feb 14;238(3):699–711. doi: 10.1007/s00221-020-05745-7 (PMC7080688; doi:10.1007/s00221-020-05745-7)
Supplement: Supplementary file 1 — Supplementary material 1 (pdf 333 KB) [file 221_2020_5745_MOESM1_ESM.pdf]

The role of acceleration and jerk in perception of above-threshold surge motion

Ksander N. de Winkel · Florian Soyka · Heinrich H. Bülthoff

Appendix 1

A possible limitation of this study is that jerk was manipulated by varying the duration of sinusoidal acceleration/deceleration peaks. By doing so, velocity (and distance, for that matter) was varied as well. It is possible to avoid this confound by defining motion profiles where acceleration/deceleration pulses are triangular[1,2]. However, because the frequency content of such motions would be similar (1Hz and above), we do not expect qualitatively different results. This is supported by simulations: we defined a set of 1s motion profiles with triangular acceleration/deceleration pulses with a fixed duration of 250ms. The amplitude of the peak was varied to achieve different maximum accelerations (i.e., $A_{max} = [0.5, 1, 1.5, 2, 2.5] m/s^2$; the timing of the peak was manipulated to achieve different maximum jerk values (i.e.,

Ksander N. de Winkel
Max Planck Institute for Biological Cybernetics
Max-Planck-Ring 14
72076, Tübingen Baden-Württemberg, Germany Tel.: +49-7071-601201
Fax: +49-7071-601616
E-mail: ksander.dewinkel@tuebingen.mpg.de
ORCID: 0000-0003-0534-2723

Florian Soyka
Max Planck Institute for Biological Cybernetics
Max-Planck-Ring 14
72076, Tübingen Baden-Württemberg, Germany Tel.: +49-7071-601201
Fax: +49-7071-601616
E-mail: florian.soyka@tuebingen.mpg.de

Heinrich H. Bülthoff
Max Planck Institute for Biological Cybernetics
Max-Planck-Ring 14
72076, Tübingen Baden-Württemberg, Germany Tel.: +49-7071-601201
Fax: +49-7071-601616
E-mail: heinrich.buelthoff@tuebingen.mpg.de
ORCID: 0000-0003-2568-0607

$J_{max} = [20, 30, 40, 50, 60]m/s^3$. The corresponding timing values for the peak were calculated as A_{max}/J_{max}). These motions were processed using the Linear Time-Invariant systems model specified in [3]. The profiles and model output are shown in Figure 1. We then took the maximum of the absolute value of

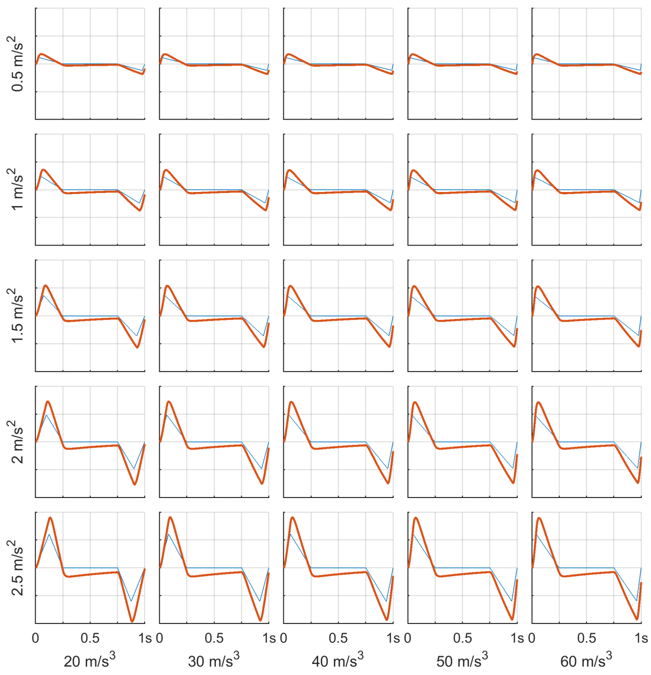

Fig. 1 Triangular motion profiles (thin blue lines) and LTI model output[3] (thick orange lines). Note that the scaling on the y-axis is arbitrary.

the model output as a simulated representation of perceived intensity (R, ψ), and fitted regression models that included only main effects for acceleration and jerk (Figure 2, left panel), or main effects and their interaction (Figure 2, right panel). For the main-effects model, the regression coefficients were $\omega_A = 47.589, \omega_J = -0.055$, which corresponds to standardized coefficients $\omega^*_A = 65.940, \omega^*_J = -0.004$, and relative weights $\tilde{\omega}_A = 0.999, \tilde{\omega}_J = -0.000$.
For the model with an interaction term, the coefficients were $\omega_A = 49.879, \omega_J = 0.031, \omega_{AJ} = -0.057$, which corresponds to standardized coefficients $\omega^*_A = 69.113, \omega^*_J = 0.002, \omega^*_{AJ} = -0.002$, and relative weights $\tilde{\omega}_A = 0.999, \tilde{\omega}_J = 0.000, \tilde{\omega}_{AJ} = -0.000$.
These simulations indicate that for triangular profiles with this frequency content, perception would be driven by acceleration.

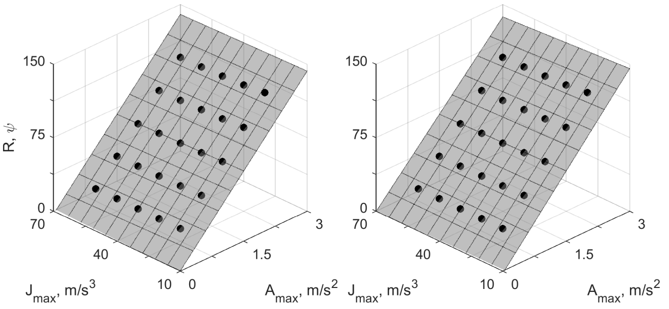

Fig. 2 Regression model fits to the peak values of the LTI model outputs shown in Figure 1. The left panel shows a regression model with main effects of acceleration and jerk only; the right panel shows a regression model that additionally includes an interaction term. Note how the perceived intensity R, ψ (arbitrary scaling) varies with acceleration A_{max} only.

References

1. G.P. Siegmund, B.E. Heinrichs, D.D. Chimich, A.L. DeMarco, J.R. Brault, Accident Analysis & Prevention **37**(2), 275 (2005)
2. G.P. Siegmund, J.S. Blouin, The Journal of physiology **587**(8), 1829 (2009)
3. F. Soyka, P. Robuffo Giordano, K. Beykirch, H.H. Bülthoff, Experimental brain research **209**(1), 95 (2011)
